# The barriers to initiating lung cancer care in low-and middle-income countries

**DOI:** 10.11604/pamj.2020.35.38.17333

**Published:** 2020-02-12

**Authors:** Buhle Lubuzo, Themba Ginindza, Khumbulani Hlongwana

**Affiliations:** 1 Discipline of Public Health Medicine, School of Nursing and Public Health, University of KwaZulu-Natal, Durban 4041, South Africa

**Keywords:** Barriers, lung cancer, low-and middle-income countries

## Abstract

Lung cancer in low-and middle-income countries is the leading and the second leading cause of cancer deaths in males and females, respectively. This, in part, is due to late presentation of patients in health facilities and late diagnosis, thereby compromising the effectiveness of treatment and resulting in poor treatment outcomes. Investigating patients’ late presentation to health facilities and late diagnosis, as barriers to achieving good treatment outcomes, is an important step towards improving the existing pathways of care. Therefore, the aim of this paper is to critically review the published and unpublished literature, including government reports on lung cancer care, with regards to the barriers to patient access, referral, diagnosis and treatment in low-and middle-income countries. The emphasis is on access point and the primary care continuum. This review has been packaged into themes in order to efficiently inform researchers and cancer health professionals, on the existing gaps necessary for developing appropriate intervention strategies and policy guidelines. This review has revealed that the timeous and correct diagnosis of lung cancer enables lung specialists to engage on options for improved patient care. Currently, there are variations in lung cancer management in low-and middle-income countries. Many of the factors impacting on health care outcomes are a function of patient circumstances and/or understanding, leading to delays in presentation to health facilities. Factors pertaining to individual patient circumstances are further compounded by inefficiencies within the health care system. Therefore, limited health system capacities and competing health priorities in these settings require action.

## Introduction

Cancer morbidity and mortality is increasingly becoming a major public health problem and it is the second leading cause of death worldwide [1]. While the overall incidence of cancer is lower in low-and middle-income countries (LMICs) compared with high-income countries (HICs), total cancer-related mortality is significantly higher in LMICs [2]. Approximately, 8 million new cancers were detected in the less developed regions of the world in 2012, and 5.3 million of them died of the disease [3]. Survival from cancer is strongly associated with geography, and patients in LMICs perform worse than those living in upper middle-income and HICs [4]. Therefore, almost 70% of all cancer deaths occur in LMICs [5]. The World Health Organisation’s (WHO) projections indicate that global annual estimates will increase to 29.5 million new cancer diagnoses and 16.5 million cancer-related deaths, by the year 2040 [2, 6, 7], unless drastic interventions are implemented. In both sexes combined, lung cancer is the most commonly diagnosed cancer (11.6% of the total cases) and the leading cause of cancer death (18.4% of the total cancer deaths), closely followed by female breast cancer (11.6%), prostate cancer (7.1%), and colorectal cancer (6.1%) [6]. Lung cancer mortality is high among men, and most deaths occur in LMICs of Eastern Europe, Western Asia, Northern Africa, Eastern and South-Eastern Asia [8]. Highest lung cancer incidence was reported in South Korea China, Turkey, Singapore and Philippines [9, 10]. An estimated 774,323 new cases were reported in China with a mortality of 690,567 people where China accounted for almost half of the total cases of lung cancer worldwide [11]. In Africa, lung cancer is the fourth most common cancer among men with approximately 39,300 and 37,700 new lung cancer cases and annual mortality, respectively [11, 12].

In 2017, only 26% of low-income countries reported having pathology services available in the public sector [13], in contrast to more than 90% of HICs reporting the availability of treatment services [13]. Effective pathways of care in the management of cancers, including lung cancer, is key to achieving quality outcomes and comfort of knowing that patients are receiving the best care [14]. However, in LMICs, studies have reported several barriers to achieving optimal health outcomes enjoyed by the HICs [3, 15-23]. In addition, socio-cultural and economic difficulties, weak health systems and poor knowledge deter many in LMICs from accessing appropriate cancer care services [16, 24-29]. While these challenges are substantial, they are not insurmountable, but the starting point is recognizing the magnitude of the problem [25]. Lung cancer patients in LMICs are bearing the effect of, not only the disease, but also the health systems. Raising awareness of risk factors and screening services for cancer in the population, especially lung cancer, along with improved access and quality of health care are key to enhancing the chances of cure and survival [27]. Accordingly, the aim of this review was to identify and assess gaps in the optimal management of lung cancer in LMICs, with a view to highlight key factors hindering the delivery of best practice for lung cancer care. This review was achieved through identifying and synthesizing the best available documented evidence on the effectiveness of lung cancer management to inform decision-makers and stakeholders on the magnitude of the problem.

## Methods

This review was conducted through a thematic analysis of the literature on the cancer care. The review is conceptual and focuses on the patterns of issues captured in research papers and other documents included in this review. The approach used in this review borrowed from the phenomenology design, which is best suited for exploring the lived experiences of people experiencing a particular phenomenon [17]. In this case, the research phenomenon was barriers to cancer care using a case study of lung cancer and the unit of analysis was research reports rather than the direct individuals who experienced the phenomenon. As a result, other studies using the same phenomenon, but different case study were included. The review was primarily focused on identifying the barriers to lung cancer patient access, investigations and referral points in LMICs. A wide variety of studies were reviewed including; qualitative, mixed method, literature reviews, government reports and research articles in order to understand the essence of phenomenon as seen through the eyes of the patients and healthcare workers and as documented by researchers who investigated the phenomenon. Existing clinical guidelines were reviewed for relevance to the study. The studies retrieved during the literature search were examined for relevance against the review objectives. No restriction was placed on the year of publication for the included reports. We limited the search to articles published in the English language. Literature included in this review was sourced through PubMed, Science Direct, Google scholar and World Health Organization (WHO) library databases search engines, using keywords, such as, lung cancer; Africa; low-and middle-income countries; cancer care; access barriers; management; patient access; primary care delays. The search yields were transferred to Endnote, version X8. We retrieved articles that specifically related to the phenomenon in LMICs. We adopted operational definitions of LMICs from the World Bank [30].

## Current status of knowledge

Literature is consistent in positioning access to comprehensive quality health care services as an important factor for promoting and maintaining health, preventing and managing disease, which translates into the timely use of health services to achieve the best health outcomes [8, 16, 21-29, 31-39]. For most patients, lung cancers are diagnosed at a late stage, thereby rendering cancer survival poor [14, 38-40]. Adequate knowledge about the disease, accessing or accessibility of a facility where relevant health care services are provided, clear and appropriate initial investigations and referral procedures to early diagnosis, staging and treatment, are required. Diagnosis and treatment aimed at detecting, prolonging, and improving the quality of life after the diagnosis of cancer is confirmed by the appropriate, accessible, diagnostic and treatment facilities. The most effective and efficient treatment is linked to early detection and consequently, early detection is directly correlated to the time patients seek help or can access health facilities [8, 21, 22, 24, 25, 27, 28, 31, 36]. With regards to timeliness, it is incumbent upon the patient to seek care and the health care system’s ability to timeously provide health care as soon as the need is recognized.

Studies show that cancer has led to a high health burden in the public sector where the management of lung cancer has been associated or reportedly having poor health outcomes in LMICs [6, 9-12, 14, 38-40]. In many cases, however, the varying health outcomes are symptoms of differences in the quality of care; including, timing of diagnosis, type of diagnostic methods used, disease staging, initiation and type of treatment [14, 38-40]. Recognising, analysing and reporting these barriers are precursors to quality improvement and the design and evaluation of effective corrective interventions to existing processes of lung cancer care [41]. In the following sections the presentation is divided in three superordinate themes, namely; (I) the patient interval, (II) cultural and societal differences, and (III) the health services context. These themes describe different aspects or intervals where the barriers are resulting from. The first describes the barriers to care from the symptom knowledge and/or onset within the patient’s interval. The following theme appreciates the diverse communities in these countries and therefore, describes the barriers to timely care resulting from the physical and social environment of patients, from different cultural, political and societal beliefs. Finally, the third theme relates to the barriers to care presenting at or within the health services context, at primary care level. The patient interval theme included three sub-themes: recognition of lung cancer symptoms, educational level and knowledge about lung cancer and, socio-economic status and place of residence. The second theme included three sub-themes: alternative medicine and illness representation, language and communication, and stigmatisation. The last theme included two sub-themes: screening services and poor referral procedures.

### PATIENT INTERVAL

**Recognition of lung cancer symptoms:** most studies now concur that the starting point for most people in seeking health, is the recognition of symptoms [8, 27, 34, 37]. In South Africa, more than 90% of patients with lung cancer have symptoms at presentation to health facilities [42], and cough is the most frequent presenting symptom. Lung cancers usually grow for years before clinical presentation and in addition, because a chronic “cigarette cough” is common in smokers, it may not seem serious until more advanced symptoms, such as blood tinged sputum occur [14, 43]. This can be further complicated by other medical conditions presenting with similar symptoms. Supporting this assertion, a study conducted in the United Kingdom on the influence of social factors on health-seeking delays for people with lung cancer, found that these delays were as a result of individuals who fail to act on symptoms, either due to fear, lack of knowledge or simply not regarding symptoms as serious enough to warrant action [44]. The fact that lung cancer symptoms are similar to that of TB, does not make things any easier for the healthcare workers as well, who often put lung cancer patients on anti-TB treatment, resulting in referral delay to oncology specialists [26-28, 39, 40, 45, 46]. Lack of knowledge on lung cancer symptoms deters patients from health-seeking, necessitating a need for public awareness campaigns to improve early health-seeking behaviour [44, 45, 47].

**Educational level and knowledge about lung cancer:** english as a default medium of communication that drive education and awareness, excludes the majority of uneducated individuals, most of whom live in the rural areas and are from the LMICs [14, 25, 39]. The shortcomings of the healthcare services and community messaging strategy lead to many patients seeking treatment late [48-50]. This is partly due to poverty and educational level, but largely to the fact that most people, in LMICs, don’t know that lung cancer can be treated [21]. Preventing lung cancer from occurring in the first place is the most definitive way to lessen the burden of cancer [50]. However, this requires knowing something about the causes or risk factors associated with it [50]. Unsurprisingly, most of the cancer literature is from HICs which may not be applicable to LMICs [50]. This is further supported by the South African Department of Health Policy, stating that mixed and often poorly understood written messages communicated primarily in English language, marginalize illiterate people from these settings [48, 49]. On the other hand, denial of the risk and avoidance of the unpleasant knowledge are also factors that delay and/or stop patients in seeking help early. Reasons for diagnostic delay are complex and multifactorial and solutions include community initiatives to educate and resource the at-risk population [18, 44, 47, 51]. In short, all people affected by lung cancer must be empowered with appropriate knowledge [16, 38] for better prevention methods. Public health awareness of the risk factors that cause lung cancer and the importance of avoiding or stopping smoking and banning asbestos should be clear [26].

**Socio economic status and place of residence:** it is evident that poverty is a major problem in LMICs and it is closely linked to ill-health, limited access to health services, as well as lack of opportunity to lead a healthy life [52]. According to Jobson, societal poverty may impede their capacity to seek healthcare, even when the needs are obvious, and services are available [27, 52]. In some of the LMICs, health services and health costs have risen considerably, creating a health divide between the rich and the poor, where those who can afford receive good quality care and those who cannot afford are excluded [53, 54]. For most people without access to health, there is a terrible paradox: “poverty exacerbates poor health while poor health makes it harder to get out of poverty” [54]. Socio-economic status (SES) largely determines a person’s place of residence and access to health care [55]. People who have higher incomes and health insurance are more likely to get tests that can detect lung cancer early and get the right treatment [32, 36]. Living in rural areas is associated with timely and comprehensive care and support [27, 32, 47]. In the case of KwaZulu-Natal, for example, all three public hospitals with oncology units are located in two cities, namely: Durban and Pietermaritzburg [27]. The burden of travel from a patient’s residence to healthcare providers is an important issue. The necessity for repeated visits for lung cancer diagnosis and treatment on an outpatient or an inpatient basis makes distance an issue with which the patient with cancer must manage during the care course [27, 32].

### CULTURAL AND SOCIETAL DIFFERENCES

**Alternative medicine and illness representation:** there is a conventional view that cancer evokes great anxiety and fear in the patient and significant others. Significant others always have “why” questions [42], but all scientific explanations answer the question “how”, but not “why”. It is the quest to find answers to “why” question that people consult sangomas, prophets and other health providers [42]. As a result, healthcare professionals grapple with meeting the expectations of diverse communities, with different cultural, religious, social and political beliefs. Culture has an influence on one’s attitudes and beliefs, in turn affecting one’s understanding of health, disease and cure [55, 56]. Illness representations may be a barrier to early diagnosis of lung cancer and adhering to treatment. Illness representations refer to a person’s beliefs and expectations about their illness [57]; for example, the cause of the cancer and these beliefs influence responses to illness and health care. Healthcare professionals must be sensitive to various cultural issues to avoid cultural blindness. A book written from a South African perspective by a team of oncology specialists concludes that healthcare professionals need an in-depth understanding of the patients’ cultural and religious beliefs [42]. It is only with this knowledge and understanding that they can truly communicate with and assist the sufferers [42].

**Language and communication:** language and communication are an important barrier for non-English speaking and other patients where the use of medical jargon, in particular, is noted as problematic. Similar to other health conditions, people with lung cancer and their carers are likely to be overwhelmed with complex medical information at a time when their thought-processing is still impaired by the stress of their illness [21]. Western-style health care starts and ends with the anatomical unit, the body of the sick person [42], where the family is usually neglected in this process and patients become isolated in the strange, sterile hospital environment, with very little communication from the providers and a few visitors or none at all if they come from far away [42]. Communication challenges by oncology nurses and oncologists have been documented in a study conducted by Watts, suggesting a formal training for health professionals to develop culturally competent communication, has been recommended to deal with patients from minority backgrounds [29].

**Stigmatisation:** stigma is relationship and context-specific where a specific attribute is associated with a negative evaluation that may lead to negative treatment or discrimination and self-fulfilling prophecies, stereotype activation, and identity threat. With lung cancer, health-related stigma is closely connected to beliefs about lung cancer causation and prognosis [58]. The association between lung cancer and smoking results in perception that lung cancer is self-inflicted, and affected patients have themselves to blame, thereby delaying treatment-seeking or even disclosing their condition to significant others [59]. Health professional behaviours were also described as blocking support of lung cancer patients, through the delay in offering appropriate referral points, due to, among other things, poor knowledge about lung cancer [25, 27, 31, 39, 42]. The psychological impact of having lung cancer include feelings of guilt and depression, which has reportedly been the same for both smokers and never smokers [59]. Promotion of right to health can potentially affect patients’ health-seeking behaviour and the provision of treatment options by health providers.

### HEALTH SERVICES CONTEXT

**Screening services:** this literature review found that early detection of cancer has held great promise and intuitive appeal in the medical community for well over a century. “Delayed presentation for cancer is the norm in many LMICs and within low-resource or geographically remote regions in upper-middle-income countries. This delay exists for a variety of structural, equity, and socio-cultural reasons” [36]. Early diagnosis might also be achieved through the provision of screening services at easy to access settings [23, 36, 46]. Screening involves looking for cancer before a person has any symptoms and the aim is to prevent cancer deaths and improve quality of life by finding cancers early and by effectively treating them [35]. Lung cancer in LMICs lack effective screening tools for early disease detection [27]. Implementation of lung cancer screening is challenging; therefore, organized lung cancer screening is practically non-existent in LMICs [8]. Lung cancer screening is largely restricted to HICs in spite of high prevalence of lung cancer cases even in LMICs [8, 35, 36]. These countries have a very high incidence of pulmonary tuberculosis and other chest infections [60-62]. Therefore, misdiagnosis is a major concern [8, 17, 26, 27, 34-36, 60, 62], which necessitates the need for screening services at primary level healthcare in these settings.

**Poor referral procedures:** inconsistent, delayed and incomplete communication amongst the different health care teams, particularly between primary and specialist care are key barriers to the delivery of coordinated patient care. Studies put forward that the increasing number of patients accessing cancer services compromises the quality of care and support health professionals provide to their patients [8, 21, 27, 28, 37, 40, 63]. Problems relating to incomplete transfer of medical information from primary care to specialists and back again are well-documented [16, 21, 27-29, 32, 42, 52]. Earlier access to lung cancer specialist care improves survival, highlighting the need for streamlined patient referral. To standardize patterns of lung cancer care and improve clinical outcomes, guidelines for optimal timing of diagnosis and treatment of lung cancer should be implemented [25, 27, 28, 31, 32, 42]. Additionally, because most lung cancer patients in the LMICs have easy access to primary healthcare facilities, nurses and doctors within these facilities should be trained on lung cancer, including the screening protocols, because professionals at these lower level facilities are not sufficiently equipped and trained, resulting in substandard performance in early diagnosis and referral [8, 12, 15, 21, 25-28, 31, 32, 35, 37-40, 47, 60, 62-64].

**Figure 1:**
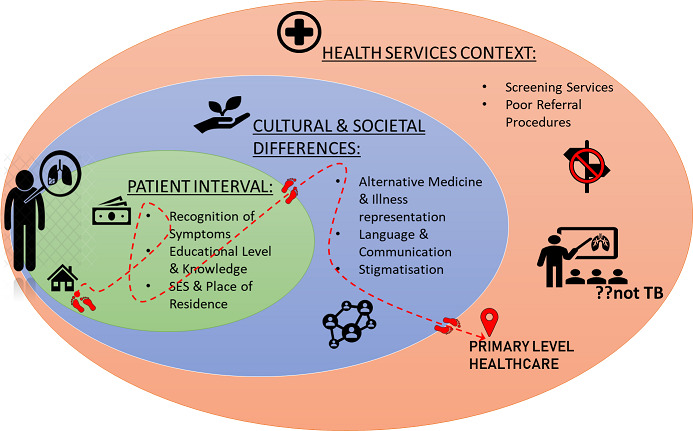
Summary of key findings made from the review according to thematic areas

It has been suggested that, well-developed cancer awareness campaigns, education, and training of health care staff regarding the possibilities of missed diagnoses and their effects on an individual patient’s mortality and morbidity can only decrease the incidence of this important health care issue [25, 27, 31, 39, 42]. Nevertheless, there remains a need to properly allocate health care resources in primary care in LMICs to avoid missed or delayed diagnoses, and that issue also highlights the difference in cancer care between HICs and LMICs [25, 27, 31, 42, 60, 62]. Therefore, streamlining the pathways of lung cancer care is key to achieving early detection of lung cancer. However, pathways of care should be both convenient and understandable to patients and healthcare professionals alike. The findings presented in this paper can be summarized through the following illustrative diagram (Figure 1): we present the results in thematic areas; the main themes being: ‘Patient Interval‚ (green), ‘Cultural and Societal Differences‚ (blue), and ‘Health Systems Context‚ (red) with their respective subthemes/ barriers to early access to lung cancer care. The patient is positioned as cutting across themes/barriers as this affects and prolong the time in which the patient reaches the primary healthcare level. As mentioned above, the red-dotted line illustrates the patients’ journey to primary healthcare, passing through the presented barriers.

## Conclusion

The outcome of this review clearly showed that micro and macro factors, such as socio-economic status and health systems affect lung cancer care in LMICs. These factors are interrelated and complex. In the South African context, lung cancer diagnosis is confirmed at a tertiary level health facility, which are located in a major metropolitan area, which limit access for rural dwellers. Long waiting times to be seen by a specialist, long distances to facilities with oncology units, deter patients from accessing these services. Early diagnosis is key to the provision and management of lung cancer care. However, there are multiple factors that affect the lung cancer care continuum, especially in LMICs. This review of literature has identified and highlighted factors contributing to adverse outcomes in lung cancer in LMICs, emanating from late presentation and delays in entering specialist care. This literature review has also revealed that the research on cancer services and care in the African region and barriers to cancer care thereof, is still limited. The fight against lung cancer is further compromised by the lack of registry in some parts of Africa, low public awareness of lung cancer and absence of screening for the high-risk cases, overburdened treatment centers and insufficient financial support [26]. In short, improved access and quality of healthcare remain important and all healthcare professionals must accept that a multi-disciplinary team is required to approach the patient, family and the management of lung cancer.

### What is known about this topic

- Patients’ late presentation to health facilities, are largely due to low suspicion index of lung cancer from the health professionals and patients alike;
- Reported variations in lung cancer care management and achievement of optimal health outcomes in HICs, are currently not applicable to LMICs;
- Cancer morbidity and mortality are becoming a major public health problem with a very high percentage of all cancer deaths occurring in LMICs.

### What this study adds

- It identifies and synthesizes the best available documented evidence on access barriers to lung cancer care in LMICs, which cause delays in presentation, diagnosis and treatment of lung cancer;
- It highlights the difference in cancer care between HICs and LMICs, which are the health systems and socioeconomic issues leading to lung cancer patients in LMICs performing worse than those living in HICs;
- It strengthens the need and importance of organising and implementing lung cancer screening services in LMICs, which are currently non-existent.
